# Oral Intake of Collagen and Collagen Hydrolysate From *Takifugu bimaculatus* Attenuates Ultraviolet‐Induced Skin Photoaging in Mice

**DOI:** 10.1002/fsn3.4559

**Published:** 2024-11-20

**Authors:** Bei Chen, Shurong Lin, Xiaoyu Yang, Shuilin Cai, Kun Qiao, Haiyan Tang, Min Xu, Yongchang Su, Shuji Liu, Zhiyu Liu, Qin Wang

**Affiliations:** ^1^ School of Life Sciences Xiamen University Xiamen China; ^2^ Fisheries Research Institute of Fujian Key Laboratory of Cultivation and High‐Value Utilization of Marine Organisms in Fujian Province Xiamen China; ^3^ College of Food Sciences & Technology Shanghai Ocean University Shanghai China

**Keywords:** collagen, hydrolysate, nutricosmetics, skin photoaging, *Takifugu bimaculatus*, ultraviolet

## Abstract

With the rapid emergence of pufferfish aquaculture and processing industries, fish skin is underutilized as a byproduct of processing, leading to resource waste. In this study, *Takifugu bimaculatus* skin collagen (TBSC) was extracted by acetic acid solubilization and its physicochemical properties were analyzed. The effects of TBSC and the TBSC hydrolysate (TBSCH) on ultraviolet (UV) irradiation‐induced photoaging were investigated using a mouse model. The purity of TBSC was 90.02%. Electrophoresis and Fourier infrared spectroscopy characterization of TBSC showed that the type of collagen in TBSC was typical standard type I. The degree of hydrolysis was selected to optimize the hydrolysis conditions for TBSC. The papain enzyme dosage, temperature, pH, and hydrolysis duration of 51,000 U/g, 48.03°C, 5.35, and 4 h have been demonstrated to be the optimum hydrolysis conditions for TBSCH. Oral administration of either TBSC or TBSCH ameliorated UV‐induced skin erythema and hyperkeratosis. TBSC and TBSCH treatment increased collagen content and had an inhibitory effect on matrix metalloproteinase (MMP)‐2 and MMP‐3 expression, whereas MMP‐9 expression was significantly reversed only in the TBSCH‐treated groups. The expression of the c‐Jun protein was much lower in these groups, suggesting that TBSCH had a greater alleviating effect on collagen degradation and extracellular matrix breakdown. Therefore, it is proposed that TBSCH has the potential to be used as a nutricosmetic agent with protective attributes against UV‐induced skin damage and concurrent collagen depletion.

## Graphical abstract

Effects of *Takifugu bimaculatus* skin collagen on UV‐induced photoaging was studied. TBSC and TBSCH ameliorated UV‐induced skin erythema and hyperkeratosis. TBSC and TBSCH inhibited MMP‐2 and MMP‐3 but TBSCH decreased MMP‐9 expression. TBSCH was better at alleviating collagen degradation and ECM breakdown than TBSC, which may be developed as a nutraceutical to delay skin photoaging.

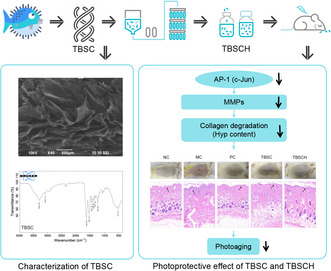

**Abbreviations**
DH: degree of hydrolysis
FTIR: Fourier transform infrared spectroscopy
GAPDH: glyceraldehyde 3‐phosphate dehydrogenase
H&E: hematoxylin and eosin
Hyp: hydroxyproline
ICR: Institute of Cancer Research
MED: minimal erythema dose
MMP: matrix metalloproteinase
RSM: response surface methodology
SDS‐PAGE: sodium dodecyl sulfate polyacrylamide gel electrophoresis
SEM: scanning electron microscopy
TBSC: *Takifugu bimaculatus* skin collagen
TBSCH: *T. bimaculatus* skin collagen hydrolysate
Td: thermal denaturation temperature
Ts: thermal shrinkage temperature
UV: ultraviolet

## 1 Introduction

Sunlight exposure, particularly to ultraviolet (UV) radiation, is the leading environmental factor responsible for numerous skin conditions such as cancer, photoaging, sunburn, and pigmentation. Solar ultraviolet radiation (UVR) that reaches the Earth surface is a combination of UVA and UVB. The latter affects the epidermis, whereas the former penetrates down into the dermis. The exploration and development of novel agents for protection and treatment against UV‐induced photodamage are critical in mitigating and rectifying the detrimental impacts of UVR on human skin.

The fish processing industry generates various by‐products, including skin, head, viscera, and bones, which are typically considered as waste. These by‐products possess a high protein content and are often utilized for producing low‐value products like animal feed, fish meal, and soil fertilizers. To increase fish value, researchers globally have focused on producing fish protein hydrolysates through the enzymatic conversion of fish proteins into smaller peptides consisting of 2–20 amino acids. These bioactive peptides exert diverse effects, including antioxidant (Sila and Bougatef 2016), antihypertensive (Abdelhedi and Nasri 2019), immunomodulatory, and antimicrobial effects (Kang et al. 2019). Consequently, they can be used as functional food ingredients, nutraceuticals, and pharmaceuticals to enhance human health and prevent diseases.

Particularly, marine collagen and collagen peptides show various skin‐protective effects owing to their antioxidant, tyrosinase‐inhibiting, matrix metalloproteinase (MMP)‐inhibiting, and anti‐inflammatory activities, whereby they can presumably be used for the development of photoprotective foods or cosmetics (Venkatesan et al. 2017). Hou et al. (2012) demonstrated that collagen polypeptides extracted from cod skin exerted UV‐protective effects by boosting immune responses, decreasing moisture and lipid loss, enhancing antioxidant properties, suppressing the accumulation of glycosaminoglycans, and aiding in the restoration of endogenous collagen. Similarly, gelatin and gelatin hydrolysates from salmon skin protect hairless mice against photoaging by relieving oxidative stress, reducing oxidative damage, maintaining collagen matrix homeostasis, and enhancing the immune system (Chen et al. 2016). Moreover, Zhuang et al. (2009) and Fan, Zhuang, and Li (2013) demonstrated that collagen and collagen hydrolysates from jellyfish effectively alleviated UV‐induced skin damage.

Because of its exceptional taste, pufferfish is considered one of the most delicious dishes in China, Japan, and Korea. Various functionally active peptides, such as angiotensin I‐converting enzyme inhibitory (Su et al. 2021), antimicrobial (Go et al. 2019), and antifreeze peptides (Yang et al. 2022), have been isolated from pufferfish skin. However, no studies on active photoprotective peptides extracted from pufferfish have been reported. *Takifugu bimaculatus* is one of the major species of pufferfish cultivated along the southeast coast of China. With an annual output of 5000 tons, it is highly valued for its high economic value and delicious taste. The production of surimi products results in 200 tons/year of fish skin by‐products. This study aims to elucidate the impact of collagen and its peptides, sourced from the skin of *T. bimaculatus*, on ultraviolet radiation‐induced photoaging, utilizing the Institute of Cancer Research (ICR) mouse model to potentially demonstrate the anti‐photoaging effects, which could pave the way for the development of novel skincare products or dietary supplements for protecting against UV‐induced skin damage and photoaging.

## 2 Materials and Methods

### 2.1 Fish Skin Preparation

Specimens of *T. bimaculatus* were obtained from Hongtun Aquaculture Co. Ltd. (Zhangpu, Zhangzhou, China). The skin was peeled, washed thoroughly, and cut into small pieces (1 × 1 cm^2^). To eliminate non‐collagenous proteins, fish skin was stirred in a 0.8 M NaCl solution (1:20, w/v) for three rounds, 10 min each. After rinsing, skin was soaked in 0.1 M NaOH (1:40, w/v) for 12 h, repeated four times. Residue was defatted in 10% butanol (1:40, w/v) for 48 h, with solvent changes every 12 h. After washing with distilled water, the skin was freeze‐dried. The samples were vacuum‐sealed and stored at −20°C.

### 2.2 Preparation of *T. bimaculatus* Skin Collagen (TBSC)

The acid solubilization method was employed to prepare TBSC. For 48 h, defatted skin was steeped in 0.5 M acetic acid at 1:40 (w/v). After collecting the supernatant, the residue was extracted under the same conditions. Both extraction filtrates were mixed. NaCl precipitated collagen from the supernatant at 2.3 M NaCl in 0.05 M Tris (pH 7.5). The precipitate was obtained after 20 min of 10,000 × *g* centrifugation. The pellet was dissolved at a ratio of 1:10 in 0.5 M acetic acid and then sequentially dialyzed in 0.1 M acetic acid and distilled water at a ratio of 1:50 for 24 h each. Finally, the sample was freeze‐dried as TBSC. All procedures were conducted at 4°C.

### 2.3 Characterization of TBSC

#### 2.3.1 Amino Acid Composition

Amino acid analysis of the TBSC was performed by the SGS‐CSTS Standards Technical Services Co. Ltd. (Xiamen Branch), according to the Chinese national standard GB 5009.124‐2016. The sample was hydrolyzed using 6 M HCl. First, the hydrolysis tube was placed on ice for 3–5 min. The tube was then connected to a vacuum pump and evacuated to near 0 Pa. Next, nitrogen was injected into the tube. The process was repeated three times, and the tube was sealed under a nitrogen atmosphere. After sealing, the tube was incubated at 110°C for 22 h and then allowed to cool to room temperature. Next, the hydrolysate was filtered into a 50 mL volumetric flask and the volume was adjusted with water. Next, 1.0 mL of the filtrate was transferred to a test tube and dried under reduced pressure at 40°C–50°C. Once dried, the residue was dissolved in water, re‐dried, and finally dissolved in pH 2.2 sodium citrate buffer. Then, the sample solution was filtered through a 0.22 μm membrane and transferred to a sample bottle. Equal amounts of the amino acid standard working solution and the sample solution were injected into the amino acid analyzer (L‐8900; Hitachi High Technologies Corp., Tokyo, Japan). The amino acid concentration in the sample was determined using the external standard method based on peak area.

#### 2.3.2 Scanning Electron Microscopy (SEM) Analysis

The collagen fibers were imaged using a scanning electron microscope (JSM‐6390LV; JEOL, Peabody, MA, USA). The samples underwent gold sputtering using an SPI Module sputter coater to achieve a thin layer deposition (SPI Supplies, West Chester, PA, USA) and imaged at an accelerating potential of 10 kV.

#### 2.3.3 Sodium Dodecyl Sulfate Polyacrylamide Gel Electrophoresis (SDS‐PAGE) Analysis

The extracted collagen was subjected to SDS‐PAGE for analytical evaluation. After dissolving 10 μg of lyophilized collagen in 1× SDS Loading Dye (Beyotime Biotechnology, Shanghai, China) and heating it at 95°C for 5 min, the collagen was resolved on 7.5% polyacrylamide gels. Following electrophoresis, the gels were treated with Coomassie Brilliant Blue R‐250 for protein band visualization.

#### 2.3.4 Fourier Transform Infrared Spectroscopy (FTIR) Analysis

The infrared absorption characteristics of the collagen were obtained using a FTIR spectrometer (Tensor II; Bruker, Karlsruhe, Germany). Collagen samples were directly added to the diamond attenuated total reflectance unit of the FTIR spectrometer. The spectra were recorded with a wavenumber range of 4000–400 cm^−1^ at a resolution of 4 cm^−1^. Collagen samples from rats were used as controls.

#### 2.3.5 Viscosity Measurement

The viscosity of 5 mg/mL collagen was measured using a rotary viscometer (NDJ‐8S; Fangrui Instrument Co. Shanghai, China). The measurement was performed at an increased temperature of 12°C.

#### 2.3.6 Thermal Shrinkage Temperature (Ts) Test

The Ts test was performed with a differential scanning calorimeter (DSC 3500 Sirius; Netzsch, Feinmahltechnik, Germany). Samples were heated from room temperature to 80°C at a rate of 1 K/min. The Ts was defined as the initial value of the endothermic peak, and the shrinkage enthalpy (ΔHs) was quantified relative to the mass of the collagen matrix.

#### 2.3.7 Effects of pH and NaCl Concentration on the Solubility of Collagen

The solubility of collagen was analyzed using the method modified by Chen et al. (2019). The lyophilized collagen was dissolved in 0.5 mol/L acetic acid to attain a final concentration of 1 mg/mL. To ensure complete solubilization, the solutions were stirred at 25°C for 2 h, followed by centrifugation at 4°C to eliminate any residual insoluble matter.

To study pH effects on collagen solubility, 7.5 mL of supernatant was transferred to a tube. The pH was adjusted to 1.0–10.0, and volume made up to 10 mL with deionized water. After centrifugation at 10,000 × *g* for 10 min at 4°C, protein concentration in the supernatant was measured using the Lowry method.

To investigate NaCl's impact on collagen solubility, 5 mL supernatant was mixed with an equal volume of NaCl to a final concentration of 0%–6% in 10 mL. After centrifugation at 10,000 × *g* for 10 min at 4°C, the supernatant was analyzed for protein concentration.

### 2.4 Preparation of *T. bimaculatus* Skin Collagen Hydrolysate (TBSCH)

#### 2.4.1 Selection of Proteolytic Enzymes

TBSC was mixed at a ratio of 1:100 (w/v) with the appropriate buffer (0.1 mol/L acetate buffer for pepsin and 0.2 mol/L phosphate buffer for all other proteases) at different pH values.

The solutions were heated in a water bath to the required temperature, after which proteases were added in suitable ratios to attain an enzymatic activity of 3000 U/g. Seven proteases, namely acid proteinase, alkaline protease, neutral protease, trypsin, pepsin, flavorzyme, and papain, were used in this experiment. After 4 h of hydrolysis, the mixtures were subjected to a 5 min heat treatment in boiling water to deactivate the proteases. Subsequently, the hydrolysates were cooled to room temperature and then centrifuged at 5000 × *g* for 10 min at 4°C. The resulting supernatant was filtered using 0.45 μm syringe filters and stored at 4°C. Hydrolysates were evaluated by measuring the degree of hydrolysis (DH).

#### 2.4.2 Single‐Factor Experiments

Five independent variables were utilized in the subsequent single‐factor experiments: enzyme concentration, pH, hydrolysis temperature, hydrolysis time, and solid–liquid ratio. The initial hydrolysis conditions when papain was used included 3000 U/g enzyme concentration, pH 6.5, 45°C temperature, incubation for 3.0 h, and solid/liquid ratio of 0.1% (w/v). The ranges of tested variables were: enzyme concentrations of 3000, 15,000, 27,000, 39,000, 51,000, and 63,000 U/g; pH of 3.5, 4.5, 5.5, 6.5, 7.5, and 8.5; hydrolysis temperature of 40°C, 45°C, 50°C, 55°C, and 60°C; hydrolysis time of 0.5, 1.0, 1.5, 2.0, 2.5, 3.0, 3.5, 4.0, 4.5, and 5.0 h; and solid–liquid ratio (w/v) of 0.05%, 0.10%, 0.20%, 0.40%, 0.80%, and 1.60%. The values of all other variables were fixed while determining the optimum level for the single variable under manipulation.

#### 2.4.3 Response Surface Methodology (RSM)

After initial single‐factor screening, the Box–Behnken RSM design determined the optimal hydrolysis temperature, enzyme concentration, and pH. DH was selected as the response variable. Statistical design, RSM modeling, and data analysis were conducted with the Design‐Expert 8.06 software. Table 1 shows the independent variables and their level code variables. A quadratic polynomial model fit using multiple regression was used to analyze the responses obtained from each experimental design set. To determine the linear, quadratic, and interaction terms' effects and regression coefficients, an analysis of variance table was produced.

**TABLE 1:** Factors and level of response surface analysis.

Coding levels	Factors		
	Hydrolysis temperature (°C)	pH	Hydrolysis enzyme concentration (U/g)
−1	45	3.5	27,000
0	50	4.5	39,000
1	55	5.5	51,000

### 2.5 Experimental Animals and Grouping

Male ICR mice, aged approximately 6–7 wk., were purchased from the Liaoning Changsheng Biotechnology Co. (Liaoning, China). The Center for Experimental Animals (Wuhan, China) Welfare Committee approved all animal experiments conducted according to standard guidelines for animal care. Throughout the study, food and water were supplied to the mice ad libitum. Following a 1 wk. acclimation period in their cages, mice were randomly assigned to five groups of eight mice each (Table S1; *n* = 40). Anesthesia was applied as ether inhalation, and the long hair was removed with surgical scissors. An area of the dorsal skin (2 × 3 cm) was then shaved using a razor. Thereafter, shaving was performed as required (usually every 2 d). Sample solutions or saline (100 μL/mouse) were administered orally daily. All mice, with the exception of those in the control group, were exposed to radiation using the identical UV source.

### 2.6 UV Irradiation

Two UVB and four UVA lamps (40 W; Beijing Lighting Research Institute, Beijing, China) were used as UV sources without any filtering. The distance between the lamps and the backs of the animals was 30 cm. Preliminary measurements were made with a UV‐radiometer (Photoelectric Instrument Factory of Beijing Normal University, Beijing, China) to determine the minimal erythema dose (MED). 3.25 μW/cm^2^ UVA and 0.134 μW/cm^2^ UVB (irradiated for 20 min) were defined as 1 MED in this study. Mice were irradiated three times weekly. UV intensities were then increased by 1 MED per week until the fourth week, and then maintained at 4 MED until the eighth week.

### 2.7 Histological Examination

Fixed mouse skin samples were paraffin‐embedded and sectioned at 6 μm thickness. Epidermal hyperplasia was quantified and regular tissue examinations were conducted using hematoxylin and eosin (H&E) staining. The images were recorded at 10× magnification using a panoramic scanner (3Dhistech, Budapest, Hungary). Epidermal thickness was statistically analyzed using Image Pro Plus software.

### 2.8 Hydroxyproline (Hyp) Content Test

Hyp, a characteristic amino acid of collagen, was quantified in the skin samples using a Hyp kit (Solarbio, Beijing, China) according to manufacturer instructions. Prior to analysis, samples were subjected to hydrolysis in 6 M HCl at 100°C for 3 h.

### 2.9 Quantitative Real‐Time PCR (qPCR) Analysis

Skin tissue (approximately 20 mg) was processed using an RNAprep Pure kit (Tiangen Biotech, Beijing, China) following the manufacturer protocols to extract total RNA. Reverse transcription was performed using the PrimeScript RT Master Mix (Takara Bio, Otsu, Japan) with 1 μg total RNA, followed by qPCR using a FastStart Universal SYBRGreen Master kit. The target (Mmp‐2, Mmp‐3, and Mmp‐9) and reference genes (glyceraldehyde 3‐phosphate dehydrogenase [Gapdh]) were amplified using the primers specified in Table S2. The target gene expression levels were relative quantified utilizing the 2^−ΔΔCt^ method.

### 2.10 Western Blotting Analysis

Homogenized skin sections were lysed in radioimmunoprecipitation assay lysis buffer (Solarbio) containing Pierce Protease Inhibitor Tablets (Thermo Fisher Scientific, Waltham, MA, USA). After 12% SDS‐PAGE was used to separate the protein (50 μg), 5% skim milk was used to stop the separation. Following three washes with Tris‐buffered saline with 0.1% Tween 20 Detergent (TBST), the membranes were incubated with the primary antibody at room temperature for 2 h. Following a 1‐h incubation period with the secondary antibody, the membranes underwent three to five TBST washes. The ECL Chemiluminescent Substrate Reagent reagent (Advansta, San Jose, CA, USA) was utilized to visualize the bands, which were subsequently detected by an imaging system (Tanon Science and Technology, Shanghai, China). Table S3 lists the antibodies used in this study.

### 2.11 Statistical Analysis

The statistical analysis was conducted using GraphPad Prism (GraphPad Software, San Diego, CA, USA). The significant difference between the UVB model group and the other groups was determined using Welch and Wilcoxon tests. Results were classified as non‐significant (ns) at *p* > 0.05, significant (*) at 0.01 < *p* ≤ 0.05, very significant (“**”) at 0.001 < *p* ≤ 0.01, and extremely significant (“***”) at *p* ≤ 0.001.

## 3 Results and Discussion

### 3.1 Amino Acid Profile of TBSC

TBSC is an acid‐soluble collagen with an extraction rate of 25.28%. The amino acid composition of skin collagen is summarized in Table 2. Glycine (Gly) was found to be the predominant amino acid in TBSC, aligning with the Gly‐X‐Y repeating sequence observed in the collagen molecule. Relatively high proline, alanine, glutamic acid, and Hyp contents were observed. As a peculiar amino acid in collagen, Hyp accounted for approximately 8% of TBSC. According to previous research in tiger pufferfish (Tsukamoto et al. 2013), the average conversion coefficient of Hyp and collagen was found to be 10.6. Therefore, the collagen content was calculated based on the Hyp content. The Hyp content of TBSC was determined to be 8.11%, and the purity of the extracted TBSC was 86.0%.

**TABLE 2:** Amino acid composition of *Takifugu bimaculatus* skin collagen (TBSC).

Amino acid	Content (%)
Glycine	24.28
Hydroxyproline	8.11
Proline	11.94
Threonine	2.25
Alanine	11.49
Glutamic acid	9.60
Arginine	8.78
Lysine	3.62
Leucine	2.09
Valine	2.55
Methionine	1.98
Histidine	0.76
Isoleucine	0.95
Serine	3.76
Aspartic acid	5.08
Tyrosine	0.51
Phenylalanine	1.91

### 3.2 Structural and Morphological Properties of TBSC

**FIGURE 1:**
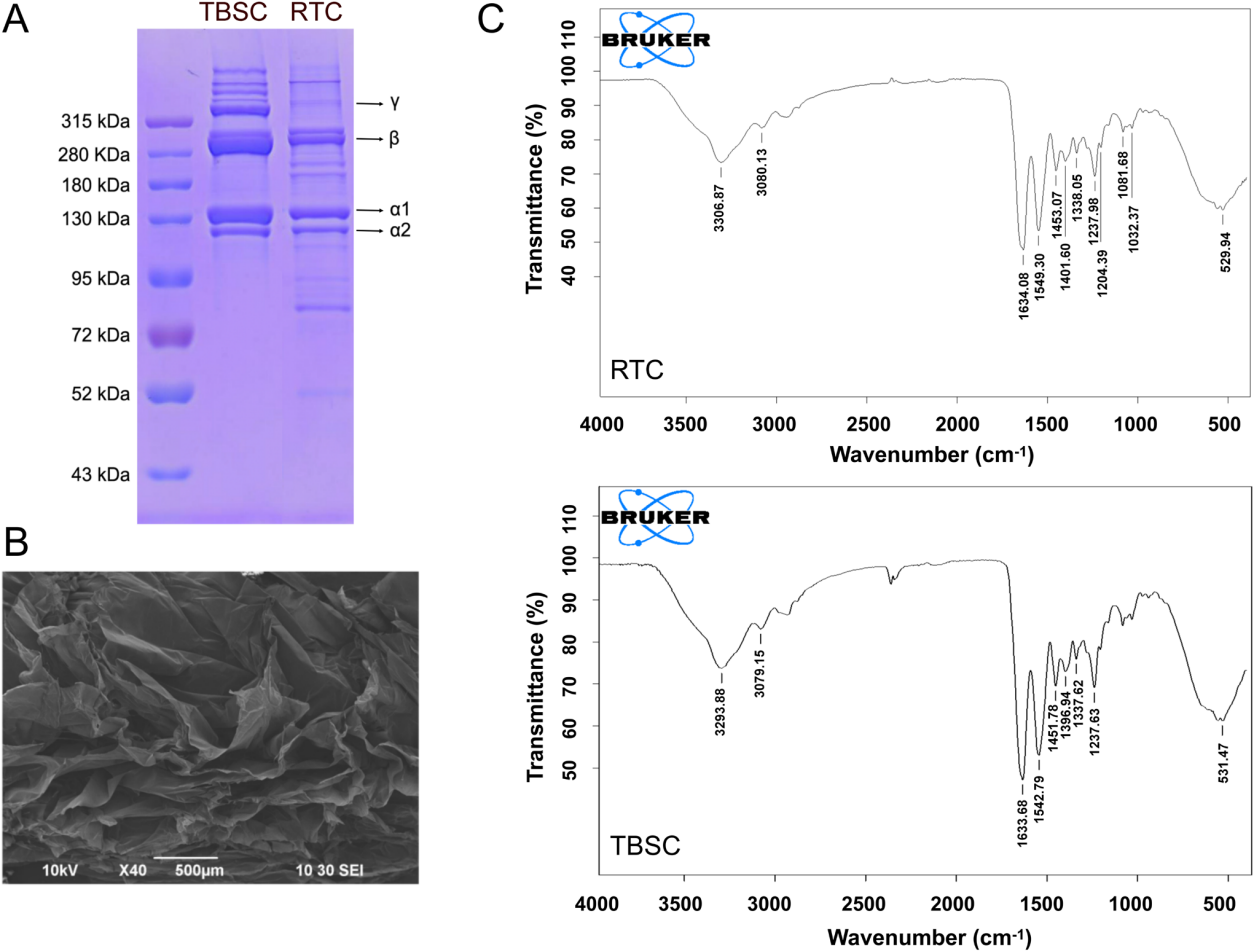
(A) Sodium dodecyl sulfate polyacrylamide gel electrophoresis (SDS‐PAGE) patterns of *Takifugu bimaculatus* skin collagen (TBSC). Lane 1, standard protein marker; Lane 2, TBSC; Lane 3, rat‐tail collagen (RTC). (B) Scanning electron microscopy (SEM) images showing the morphological features of TBSC. (C) Fourier transform infrared spectroscopy (FTIR) results for TBSC and RTC.

SDS‐PAGE revealed the subunit composition and collagen type, indicating that TBSC exhibited similarities to rat‐tail type I collagen. This collagen type consists of α‐chains (α1 and α2), β‐chains (dimer), and a small number of γ‐chains (trimer) (Figure 1A). The molecular weight (MW) of the α1‐chain (near 140 kDa) was higher than that of the α2‐chain (near 110 kDa). The band intensity of α1 was twice that of α2. Further, the results suggested that collagen from *T. bimaculatus* skin was characterized as type I collagen ((α1)2α2). The electrophoretic patterns of the TBSC were similar to those of collagen from other fish, such as giant croaker (*Nibea japonica*) (Chen et al. 2019), bigeye tuna (*Thunnus obesus*) (Ahmed, Haq, and Chun 2019), Miiuy croaker (*Miichthys miiuy*) (Zhao et al. 2018), rohu (*Labeo rohita*) (Gaurav Kumar et al. 2017), grass carp (*Ctenopharyngodon idella*) (Liu et al. 2015), and Nile tilapia (*Oreochromis niloticus*) (Sun et al. 2017).

The ultrastructure of the cross‐section of the lyophilized collagen was analyzed using SEM (Figure 1B). The micrographs showed that TBSC displayed a loose porous network structure in the 40× magnified image. TBSC also showed a dense sheet‐like film with uniform alignment. The results of SEM for TBSC were similar to those for collagen extracted from the swim bladders and the skin of the giant croaker (Chen et al. 2019) and those of the swim bladders of the Miiuy croaker (Zhao et al. 2018).

Collagen is remarkable for its application in biomedical materials. The microscopic structure of collagen determines its physicochemical properties and bio‐functionality. Previous studies have suggested that collagen possessing fibrous and sheet‐like film structures, along with interconnectivity, can be utilized for various applications, such as tissue regeneration, cell seeding, wound healing, gene expression, and for facilitating mass transport and migration (Zhao et al. 2018). Therefore, the microstructure of TBSC indicates that it can be used in different biomedical material fields.

Information about the secondary structure of TBSC was obtained using FTIR spectroscopy. The FTIR absorption spectra (4000–400 cm^−1^) and the assignment of major peaks (amide A, B, I, II, and III) of TBSC are shown in Figure 1C. The spectral characteristics of TBSC were consistent with those documented in the literature for collagen derived from other fish species.

Amide A bands were found at wavenumbers of 3293.88 cm^−1^ for TBSC, which corresponded to the stretching vibrations of N‐H groups. These vibrations are independent of backbone conformation, and highly sensitive to hydrogen bonding strength. Asymmetric stretch vibrations of −NH_3_^+^ and = C–H of TBSC were shown by amide B band positions at wavenumbers of 3079.15 cm^−1^. The amide I bands, found at wavenumbers 1633.68 cm^−1^, primarily represent the stretching vibrations of C=O and C–N groups. The frequencies in the range of 1600–1700 cm^−1^ are influenced by the conformation of the backbone and the arrangement of hydrogen bonding. Meanwhile, amide II was observed in the 1510 and 1580 cm^−1^ regions and was more complex than amide I. The in‐plane N–H bending vibrations associated with C–N stretching vibration and C–H stretching are represented by the amide II band at 1542.79 cm^−1^ and the amide III band at 1237.63 cm^−1^. The FTIR spectra of TBSC exhibited an absorption ratio of approximately 1.0 between amide III (1237 cm^−1^) and 1452 cm^−1^, thus providing evidence that the triple‐helical structure of collagen remained intact.

### 3.3 Thermal Stability and Solubility of TBSC

**FIGURE 2:**
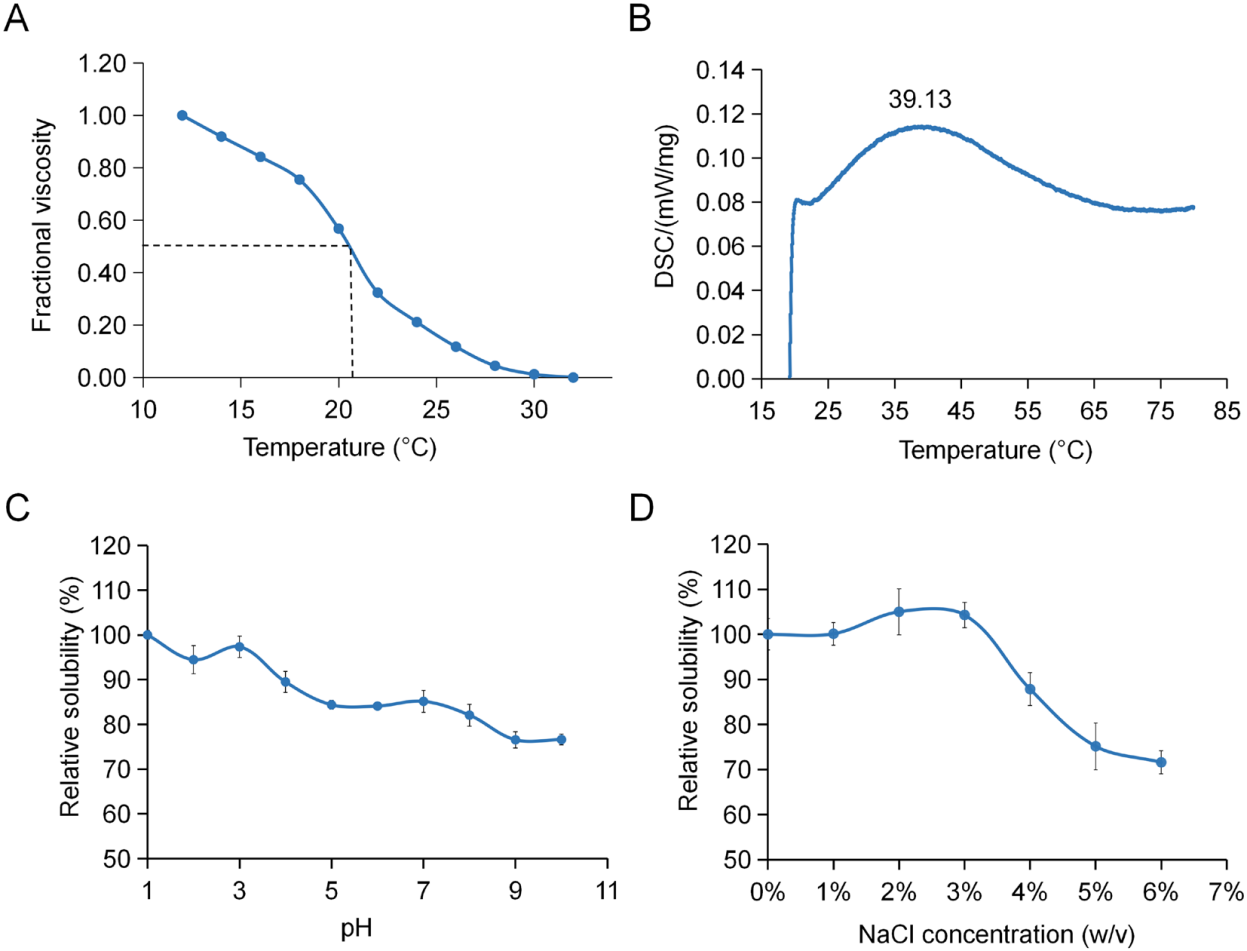
(A) Thermal denaturation curves of *Takifugu bimaculatus* skin collagen (TBSC). The denaturation temperature was determined as the mid‐point temperature at which the viscosity change reached 0.5. (B) Thermal transition curve of TBSC. (C) Solubility of TBSC at different pH values. (D) Solubility of TBSC at different NaCl concentrations.

The thermal stability of collagen was assessed through determining the temperature of thermal denaturation of the molecules in solution (thermal denaturation temperature, Td) or the temperature at which the fibers undergo thermal contraction (Ts). Increasing temperature leads to the gradual breakdown of hydrogen bonds that protect the triple‐helical structure of collagen, resulting in the production of random coil chains through thermal depolymerization. Viscometry is a widely used method to study the conformational changes in macromolecules in solution, making it a valuable tool for investigating the thermostability of collagen. Figure 2A shows the relative viscosity of the 0.5% TBSC solution, which exhibited a rapid decline as temperature increased. Its Td value was determined as 20.4°C. Compared to mammalian collagen (39°C–40°C), collagen derived from fish skin, bones, and fins shows a lower denaturation temperature (typically around 25°C–30°C for most species) and has a variable composition (Huang et al. 2011; Jafari et al. 2020; Faralizadeh et al. 2021), indicating that the helix structures of TBSC were more unstable than those of collagens from mammals. Particularly, Hyp enhances the stability of the collagen triple helix (Meyer 2019). The relatively low content of amino acids such as proline and Hyp in fish collagen contributes to its low denaturation temperature.

The thermal stability of collagen was also tested using the corresponding Ts. Heating destroys the secondary, tertiary, and quaternary structures of collagen, resulting in degeneration. Ts is the temperature at which the collagen fibers shrink to one‐third its length. The Ts value of TBSC was 39.13°C (Figure 2B). In contrast, the Ts of mammalian collagens remains consistent at approximately 65°C–67°C, whereas fish and invertebrate collagens exhibit significant variation, ranging from 35°C to 55°C. This strong variation in the Ts of fish and invertebrates reportedly correlates with Hyp content and habitat temperature (Miles and Bailey 1999).

Effects of pH and NaCl on collagen solubility were then tested. The solubility of TBSC at different pH values is shown in Figure 2C. TBSC exhibited maximum solubility under acidic conditions and showed a decreasing trend as pH increased.

The solubility of TBSC increased and then decreased with increasing NaCl concentration (Figure 2D). At low NaCl concentrations, collagen combined with Na^+^, the positive charge of collagen increased, and the repulsive force between proteins also increased, making the system more stable and increasing solubility. Conversely, collagen solubility rapidly decreased when NaCl concentration reached approximately 3% (w/v). Higher concentrations of NaCl increase ionic strength and facilitate hydrophobic interactions among protein chains, resulting in reduced collagen solubility and subsequent protein precipitation (Zhang et al. 2019).

### 3.4 Optimizing Enzymatic Conditions for TBSCH

#### 3.4.1 Selection of the Best Protease

**FIGURE 3:**
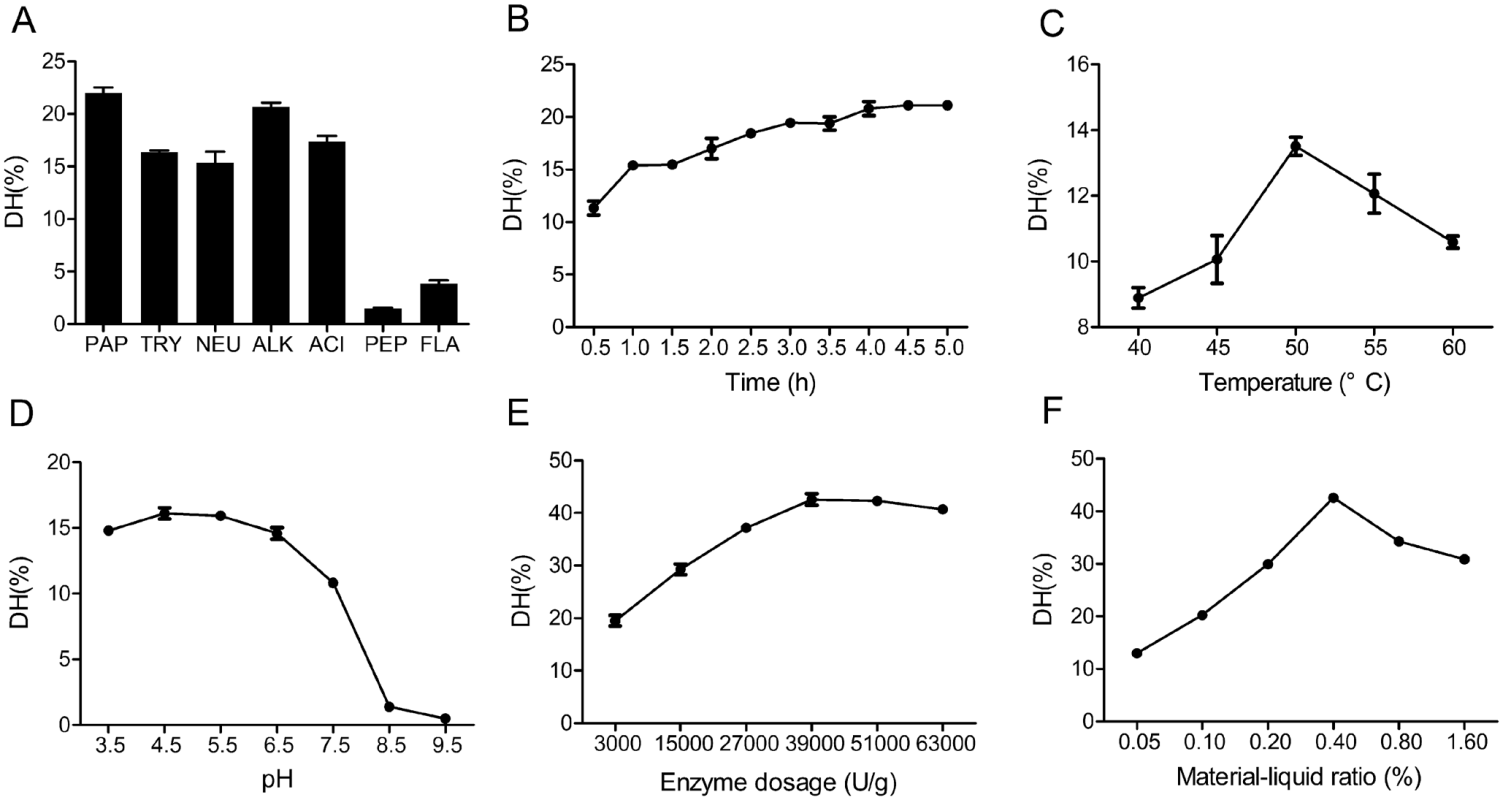
(A) Degree of hydrolysis (DH) of hydrolysates produced by various commercial proteases including acid proteinase (ACI), alkaline protease (ALK), neutral protease (NEU), trypsin (TRY), pepsin (PEP), flavorzyme (FLA), and papain (PAP). Effects of independent variables on DH: (B) hydrolysis time, (C) temperature, (D) pH, (E) enzyme dosage, and (F) material‐liquid ratio.

Several commercial proteolytic enzymes are used to hydrolyze proteins from fish by‐products (Zamora‐Sillero, Gharsallaoui, and Prentice 2018). A comparative analysis was conducted on multiple proteases to determine their efficacy in producing hydrolysates. The protease reactions were conducted using consistent solid/liquid ratios, enzyme volumes, and enzymolysis times, while ensuring optimal pH and temperature conditions for each specific enzyme. Compared to other hydrolysates, collagen had the highest DH value of 22.0% when papain was used. The average DH values for the remaining hydrolysates were as follows: 16.4% for trypsin, 15.3% for neutral protease, 20.7% for alkaline protease, 17.4% for acid proteinase, 1.5% for pepsin, and 3.9% for flavorzyme (Figure 3A). Papain is a plant proteolytic enzyme from the cysteine proteinase family (Mamboya and Amri 2012) that can generate bioactive peptides from different types of fish by‐products, such as Malabar grouper (*Epinephelus malabaricus*), Asian sea bass (*Lates calcarifer*) (Hema et al. 2017), and pangasius catfish (*Pangasius pangasius*) (Baehaki, Nopianti, and Anggraeni 2015).

#### 3.4.2 Single‐Factor Experiment

The impact of hydrolysis time, temperature, pH, enzyme concentration, and material‐liquid ratio on the DH was observed. A hydrolysis time of 0.5–5 h was evaluated (Figure 3B). The DH continuously increased and reached 20.80% at 4.0 h. No significant difference was observed in DH at 4.0–5.0 h. For hydrolysis temperature, DH of the enzymatic digest first increased and then decreased with increasing temperature, reaching a maximum of 13.51% at 50°C (Figure 3C). In the meantime, the enzymatic hydrolysate's DH peaked at pH 4.5 and sharply decreased above pH 6.5 (Figure 3D). As for enzyme concentration, DH reached a maximum of 42.57% at 39,000 U/g (Figure 3E). Another significant element that can have a significant impact on DH is the material‐liquid ratio; in this study, DH reached a maximum at a material‐liquid ratio of 0.4% (Figure 3F). These findings led us to determine that the following parameters—50°C, pH 4.5, enzyme concentration of 39,000 U/g, material‐liquid ratio of 0.4%, and hydrolysis duration of 4 h—were optimal for the protease extraction of hydrolysates.

#### 3.4.3 RSM

##### 3.4.3.1 Model Fitting and Statistical Analysis

Based on the results of the single‐factor experiments, temperature, pH, and enzyme dosage were identified as significant factors for optimizing TBSC hydrolysis. To investigate these parameters at three levels, a Box–Behnken statistical design was used, for which 17 experimental runs were required (Table 3).

**TABLE 3:** Program and experimental results of response surface analysis.

Run	Temperature (°C)	pH	Enzyme concentration (U/g)	DH (%)
1	1	1	0	41.34
2	0	0	0	40.51
3	0	0	0	43.99
4	−1	1	0	41.15
5	−1	−1	0	37.82
6	0	0	0	42.64
7	−1	0	−1	36.69
8	1	−1	0	40.06
9	1	0	−1	36.67
10	0	1	−1	37.95
11	0	0	0	44.55
12	0	1	1	46.95
13	0	−1	−1	34.97
14	0	−1	1	41.41
15	1	0	1	43.31
16	−1	0	1	45.54
17	0	0	0	42.21

The model demonstrated significance of *p* = 0.0045, suggesting its suitability for optimizing the conditions of hydrolysis (Table 4). Among the three independent variables, the p‐value of enzyme dosage (C; *p* < 0.0001) was lower than those of pH (B; *p* < 0.0151) and temperature (A; *p* < 0.9662), indicating that the enzyme dosage had a greater influence than pH or temperature on DH. A substantial connection between the experimental and projected values was verified by a *p*‐value of 0.6411 and an *F*‐value of 0.61, which indicates a lack of fit. The model's applicability was tested using the determination coefficient (*R* ^2^ = 0.9188), which showed a good correlation between the rate values predicted by the equation and those found empirically. Furthermore, the experimental values were deemed reliable, as evidenced by the low coefficient of variation (3.54%). These results suggested that the model worked adequately for predicting DH of peptide hydrolysates.

**TABLE 4:** Analysis of variance for response surface quadratic model.

Source	Sum of squares	Df	Mean square	*F*‐value	*p*	Significance level
A	4.05 × 10^−3^	1	4.05 × 10^−3^	1.923 × 10^−3^	0.9662	
B	21.55	1	21.55	10.23	0.0151	*
C	119.58	1	119.58	56.78	0.0001	**
AB	1.05	1	1.05	0.50	0.5028	
AC	1.22	1	1.22	0.58	0.4713	
BC	1.64	1	1.64	0.78	0.4071	
A^2^	6.34	1	6.34	3.01	0.1262	
B^2^	8.98	1	8.98	4.26	0.0779	
C^2^	4.21	1	4.21	2.00	0.2003	
Residual	14.74	7	2.11			
Lack of fit	4.64	3	1.55	0.61	0.6411	
Pure error	10.09	4	2.52			
Model	166.80	9	18.53	8.80	0.0045	**
Cor total	181.54	16				

Abbreviations: A, temperature; B, pH; C, enzyme dosage. *p* value indicates the significance level of * 0.05 and ** 0.01.

Multiple regression analysis was used to generate a second‐order polynomial equation that describes the relationship between DH and the three variables:

$$\begin{array}{c} Y=42.78+0.02\text{3A}+1.64B+3.87C-0.51\mathrm{AB}-0.55\mathrm{AC}\\ \;+0.64\mathrm{BC}-1.23A^{2}-1.46B^{2}-1.00C^{2} \end{array}$$

where, *Y* represents DH and A, B, and C represent temperature, pH, and enzyme activity, respectively.

**FIGURE 4:**
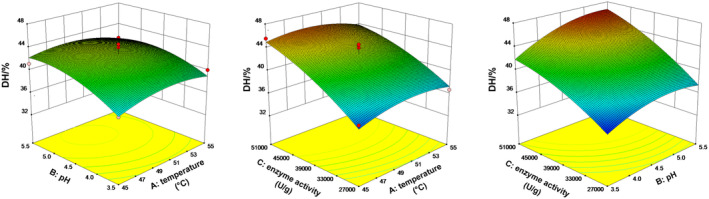
Interactive effect of extraction variables on DH.

Figure 4 shows the effects of the interaction of enzyme activity and temperature, pH and enzyme activity, and pH and temperature, on DH. Overall, the *p*‐value of each interaction was > 0.05 (*p* = 0.5028, 0.4713, and 0.4071, respectively), indicating that the interaction was not significant.

##### 3.4.3.2 Validation of the Model

A verification experiment was carried out under optimal conditions, which included an enzyme dosage of 51,000 U/g, a temperature of 48.03°C, a pH of 5.35, a material‐liquid ratio of 0.4%, and a hydrolysis period of 4 h, in order to evaluate the accuracy of the model equation. The observed mean DH was 46.79% (*n* = 3), in close agreement with the 46.72% expected value. This outcome validated the response model's correctness.

Table S4 and Figure S1 display the analyzed MW distribution of *Takifugu bimaculatus* skin collagen hydrolysate (TBSCH). With 74.3% of TBSCH falling below 8000 g/mol, the average MW was 8514 g/mol.

### 3.5 Effect of Dietary TBSC and TBSCH on Body Weight and Other Tissues

Body and tissue weights were measured in all five groups during the experimental period (Table S5). After treatment for 8 week, tissue weights were not significantly different among groups. Furthermore, no significant difference was observed in body weight among the groups.

### 3.6 TBSC and TBSCH Prevented UV‐Induced Skin Damage

**FIGURE 5:**
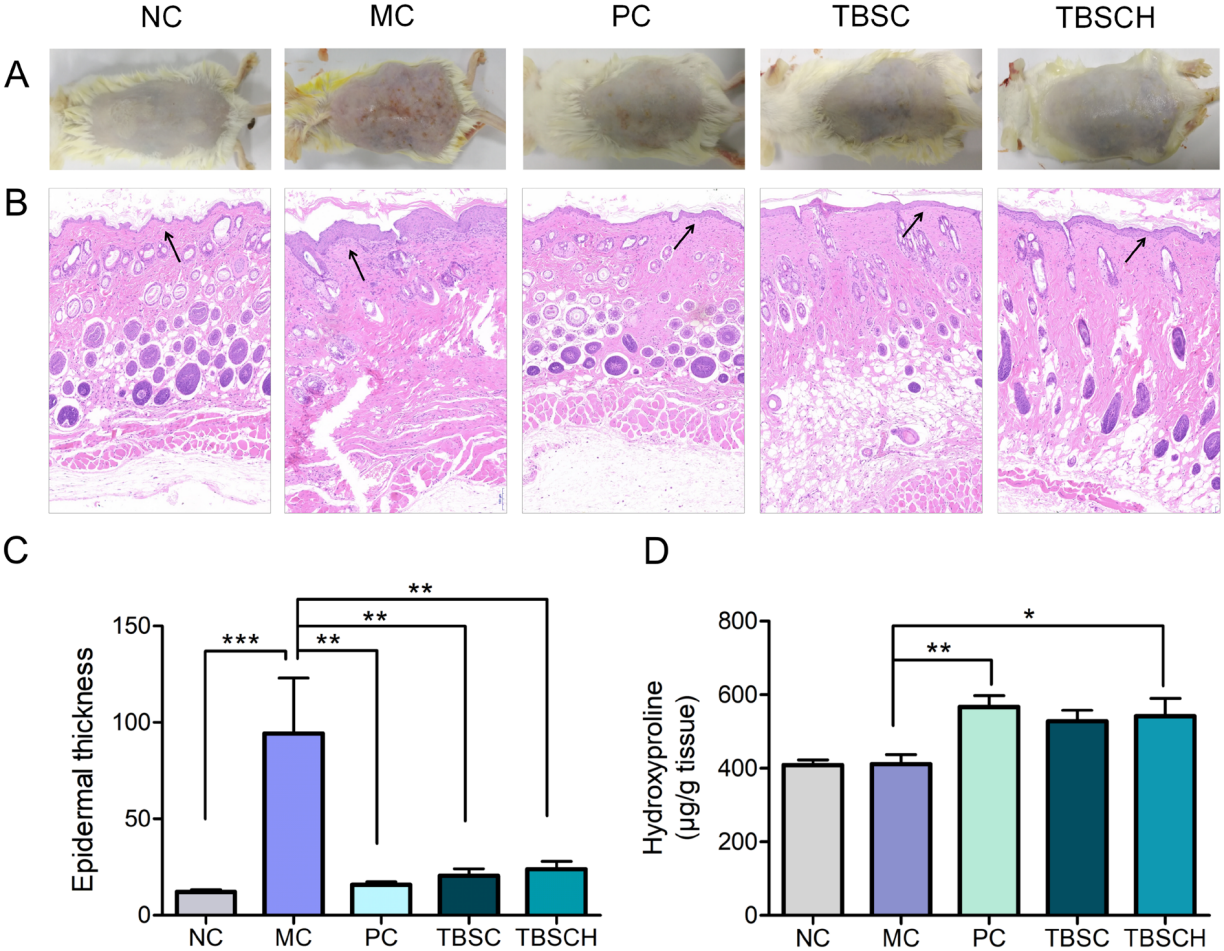
Effects of *Takifugu bimaculatus* skin collagen (TBSC) and TBSC hydrolysate (TBSCH) on skin morphology and hydroxyproline (Hyp) content of photoaged mice. Mice received saline (MC), TBSC, TBSCH, or VC (PC) for 8 wk. by oral gavage and were concurrently exposed to UV irradiation on their dorsal surface. The non‐UV‐exposed control group (NC) of mice were also treated with saline. (A) Visual appearance of different groups after the last treatment. (B) Photographs of dorsal skin sections stained with Hematoxylin and eosin (H&E). The black arrow indicates the epidermis thickness of the Institute of Cancer Research mice. (C) Epidermal thickness was measured and analyzed in each group of mice. (D) Hyp content in mouse skin. The asterisks denote significant differences of * *p* ≤ 0.05, ** *p* ≤ 0.01, *** *p* ≤ 0.001.

Images of dorsal skin and tissue sections stained with H&E for analysis are shown in Figure 5. Morphological and histopathological observations of the skin tissues of different groups showed that exposure to UV light led to noticeable skin erythema and hyperplasia. Conversely, TBSC and TBSCH ameliorated these pathological skin changes (Figure 5A). UV exposure increased epidermal thickness, known as hyperkeratosis. Epidermal keratinocytes, which are mediated by various epidermal growth factors, proliferate notably after several hours of exposure to UV (D'Orazio et al. 2013). Further, enhanced keratinocyte proliferation contributes to the accumulation of epidermal keratinocytes, leading to an increase in epidermal thickness, which in turn can provide additional protection against further UV‐induced skin damage (Scott et al. 2012). Epidermal thickness can be quantified to reflect the damage caused to the skin by UV exposure in terms of histologic features (Im et al. 2020). In this study, epidermal thickness in the mice treated with saline (MC) was uneven and thicker than that in the non‐UV‐exposed control mice (NC) (Figure 5B,C). Dietary TBSC and TBSCH ameliorated the increase in epidermal thickness caused by UV radiation in the MC group.

Oral administration of collagen or collagen peptides derived from various fish species has been reported to decrease epidermal thickness in mice exposed to UV radiation. For instance, similar efficacy in reducing skin thickness was observed in mouse models exposed to UVB radiation with collagen peptides derived from tilapia (Hou et al. 2012), and rohu (Song et al. 2023).

Additionally, Hyp is an important indicator of the skin matrix collagen. In this study, TBSCH‐treated mice had significantly higher collagen and Hyp levels compared to those in the MC group (Figure 5D). Collagen hydrolysate from chum salmon is believed to be able to increase Hyp content in chronologically aging skin owing to its effect on collagen matrix homeostasis (Liang et al. 2010).

### 3.7 Effects of TBSC and TBSCH on Suppressing Extracellular Matrix (ECM) Breakdown in UV‐Exposed Mouse Skin

**FIGURE 6:**
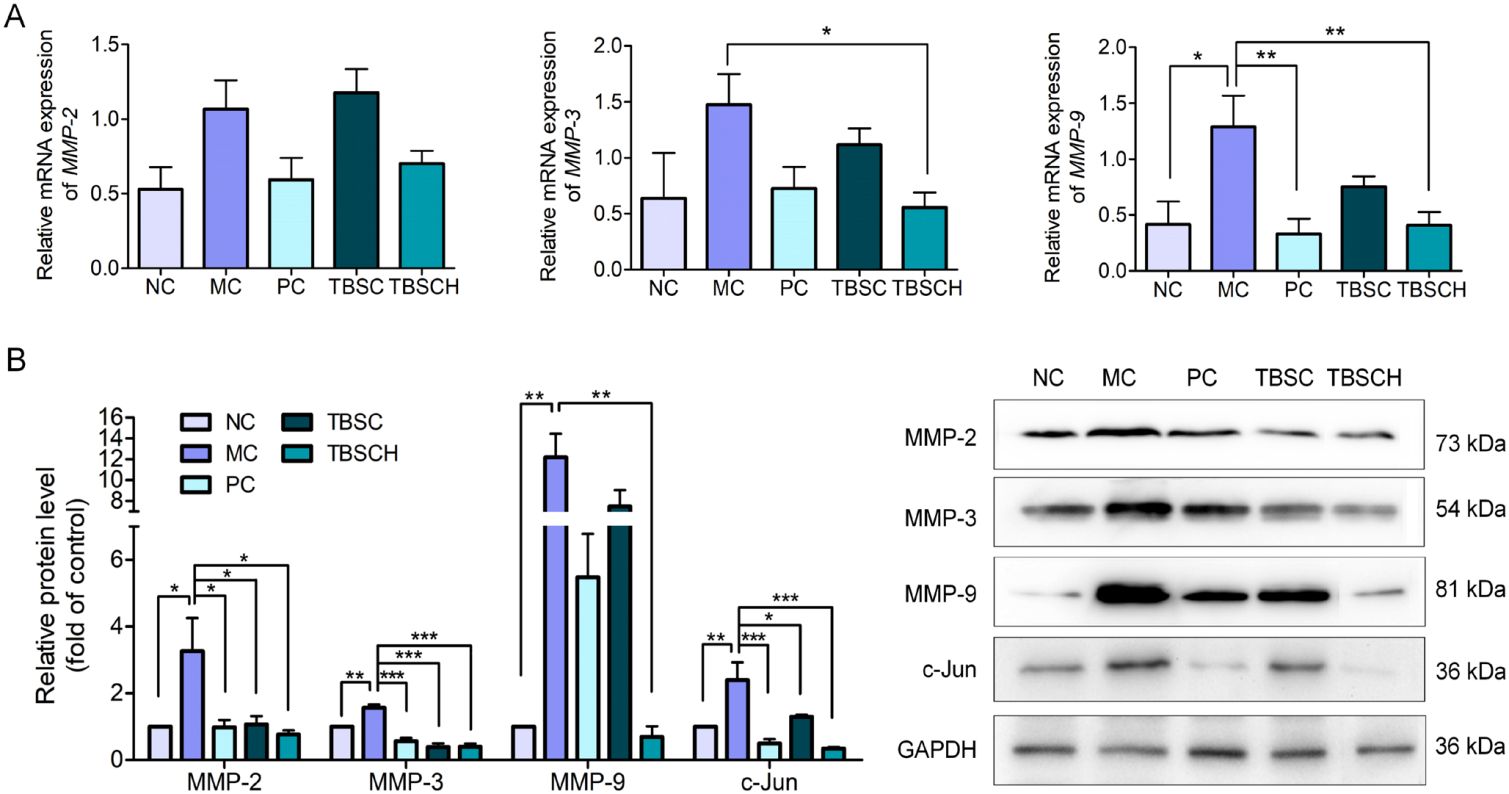
Effect of *Takifugu bimaculatus* skin collagen (TBSC) and TBSC hydrolysate (TBSCH) treatments on matrix metalloproteinase (MMP) expression in vivo. (A) mRNA expression of MMPs as assessed using quantitative real‐time PCR (qPCR). (B) Protein expression of MMPs and c‐Jun was assessed using western blotting. The protein band intensity quantification was analyzed using Quantity One software (Bio‐Rad, Hercules, CA, USA). Glyceraldehyde 3‐phosphate dehydrogenase (GAPDH) was used as an internal control for both mRNA and protein levels. The asterisks denote significant differences of * *p* ≤ 0.05, ** *p* ≤ 0.01, *** *p* ≤ 0.001.

UV radiation impacts the skin's connective tissues by increasing the expression of MMP and the enzymes that break down collagen and other extracellular matrix (ECM) proteins. (Kwon et al. 2019). c‐Jun and c‐Fos, which are elements of the activator protein 1 complex, control the synthesis of MMP. Previous studies demonstrated that treatment with collagen or collagen hydrolysate regulate MMP levels, thereby inhibiting collagen degradation (Liang et al. 2010; Pyun et al. 2012; Chen et al. 2022). The effects of TBSC and TBSCH on MMP expression in UV‐exposed mouse skin were assessed in this investigation by measuring the amounts of mRNA and protein expression using qPCR (Figure 6A) and western blotting (Figure 6B). When compared to the NC group, UV exposure significantly increased the protein production of MMP‐2, MMP‐3, and MMP‐9. However, treatment with 50 mg/kg mouse/d Vitamin C (VC), TBSC, or TBSCH significantly reduced MMP‐2 and MMP‐3 protein levels; meanwhile, MMP‐9 expression was only reversed in the TBSCH group. Compared to the TBSC‐treated group, the c‐Jun protein expression level was considerably lower in the TBSCH‐treated group. These findings suggest that TBSCH may have a stronger regulatory effect than TBSC on the expression of MMPs. The results of qPCR analysis revealed that after UV irradiation, except for MMP‐9, the mRNA expression levels of MMPs did not exhibit any significant change, but all of them tended towards upregulated expression. Further, compared with TBSC, TBSCH had a greater alleviating effect on the upregulation of MMP mRNAs induced by UV treatment.

According to Fan, Zhuang, and Li (2013), in a histological study of UV‐exposed mice, collagen hydrolysate from jellyfish umbrellas showed a much higher photoprotective ability than collagen. Moreover, Chen et al. (2016) demonstrated that gelatin hydrolysate isolated from salmon skin provided better protection from photoaging than gelatin. These findings indicate that collagen hydrolysate products have better absorption and utilization efficiencies than collagen. In a randomized controlled trial, both enzymatically hydrolyzed collagen and NC were found to significantly increase plasma amino acid concentrations over a 240‐min period. However, enzymatically hydrolyzed collagen exhibited higher absorption rate and bioavailability for glycine, proline, and Hyp, compared to NC (Skov et al. 2019).

Moreover, we also administered higher concentrations of TBSC and TBSCH (200 mg/kg/d) to the mice in this study in addition to the 50 mg/kg/d dose by gavage (data not shown). Analysis of mouse dorsal skin photographs and histological sections revealed no significant differences between high and low concentrations (data not shown). Moreover, western blotting results indicated that high‐dose TBSC and TBSCH unexpectedly diminished the inhibitory effects on MMPs and c‐jun (Figure S2). This suggests that the concentration of oral administration is crucial for the efficiency of skin resistance to UV exposure. Higher concentrations may not necessarily benefit skin repair, necessitating further research to elucidate the underlying mechanisms.

## 4 Conclusions

In this study, acid‐soluble TBSC was extracted from the skin of *T. bimaculatus*, which has been demonstrated to possess typical type I collagen characteristics. Oral administration of TBSC and TBSCH attenuated UV‐induced skin damage and collagen degradation in mice via MMP regulation. TBSCH demonstrated much higher bioactivity than TBSC, thus demonstrating greater potential as a bioactive ingredient in nutricosmetics for its beneficial effects on delaying skin photoaging.

## Author Contributions

**Bei Chen:** investigation, writing – original draft. **Shurong Lin:** formal analysis. **Xiaoyu Yang**, **Shuilin Cai**, and **Kun Qiao:** methodology. **Haiyan Tang:** investigation. **Min Xu** and **Yongchang Su:** methodology, formal analysis. **Shuji Liu**, **Zhiyu Liu**, and **Qin Wang:** methodology, supervision, writing – review and editing. All authors have read and approved the final version of the manuscript.

## Ethics Statement

All animal experiments comply with the ARRIVE guidelines and were carried out in accordance with the U.K. Animals (Scientific Procedures) Act, 1986 and associated guidelines, EU Directive 2010/63/EU for animal experiments, or the National Research Council's Guide for the Care and Use of Laboratory Animals. The animal protocol in this study was approved by Wuhan Cloud‐Clone Corp. Experimental Animal Management and Use Committee, China (IACUC permit number: IACU18‐0249).

## Conflicts of Interest

The authors declare no conflicts of interest.

## Supporting information


Data S1.

## Data Availability

Data obtained in this study are available from the corresponding author upon request.
