# Supplementary material for: Oral Intake of Collagen and Collagen Hydrolysate From Takifugu bimaculatus Attenuates Ultraviolet‐Induced Skin Photoaging in Mice
Source: Food Sci Nutr. 2024 Nov 20;12(12):10605–17. doi: 10.1002/fsn3.4559 (PMC11666967; doi:10.1002/fsn3.4559)
Supplement: Supplementary file 1 — Data S1. [file FSN3-12-10605-s001.docx]

**Supplementary Material**

**Table S1 Treatment schedule of the study**

|  | **UV radiation** | **Saline** | **VC** | **TBSC** | | **TBSCH** | |
| --- | --- | --- | --- | --- | --- | --- | --- |
|  |  |  | **mg/kg mouse/d** | | | |  |
| NC | - | + | - | - | - | |  |
| MC | + | + | - | - | - | |  |
| PC | + | - | 50 | - | - | |  |
| TBSC | + | - | - | 50 | - | |  |
| TBSCH | + | - | - | - | 50 | |  |

**Table S2 Primers used in qPCR**

|  | **Forward primer (5’-3’)** | **Reverse primer (5’-3’)** |
| --- | --- | --- |
| *MMP2* | CAGGGAATGAGTACTGGGTCTATT | ACTCCAGTTAAAGGCAGCATCTAC |
| *MMP3* | GGCCTGGAACAGTCTTGGC | TGTCCATCGTTCATCATCGTCA |
| *MMP9* | AATCTCTTCTAGAGACTGGGAAGGAG | AGCTGATTGACTAAAGTAGCTGGA |
| *GAPDH* | TCATTGACCTCAACTACAGGT | CTAAGCAGTTGGTGGTGCAG |

**Table S3 Antibody information**

|  | **Name** | **Serial Number** | **Brand** | **Dilution ratio** | **MW (kDa)** |
| --- | --- | --- | --- | --- | --- |
| Primary antibody | GAPDH | 60004 | Proteintech | 1/10000 | 36 |
|  | c-Jun | ab32137 | Abcam | 1/3000 | 36 |
|  | MMP2 | ab181286 | Abcam | 1/1000 | 73 |
|  | MMP3 | ab52915 | Abcam | 1/1000 | 54 |
|  | MMP9 | ab228402 | Abcam | 1/1000 | 81 |
| Secondary antibody | Goat Anti-Rabbit IgG HRP Conjugate | N20915 | TransGen Biotech | 1:10000 |  |

**Table S4 The molecular weight distribution of the TBSCH**

| **High Limit Mw (g/mol)** | **Low Limit Mw (g/mol)** | **Percent Mw (%)** |
| --- | --- | --- |
| 113393 | 100000 | 0.04 |
| 100000 | 80000 | 0.17 |
| 80000 | 50000 | 0.95 |
| 50000 | 30000 | 3.38 |
| 30000 | 20000 | 6.66 |
| 20000 | 10000 | 10.68 |
| 10000 | 8000 | 3.87 |
| 8000 | 3000 | 50.28 |
| 3000 | 2000 | 11.65 |
| 2000 | 1000 | 8.37 |
| 1000 | 500 | 3.07 |
| 500 | 203 | 0.89 |

**Table S5 Body weight and tissue weight of mice**

|  | **Weight**  **（g）** | **Spleen/**  **weight（%）** | **Thymus/**  **weight（%）** | **Heart/**  **weight（%）** | **Liver/**  **weight（%）** | **Kidney/**  **Weight**  **（%）** |
| --- | --- | --- | --- | --- | --- | --- |
| NC | 42.00±2.00 | 0.39±0.10 | 0.19±0.06 | 0.51±0.06 | 3.99±0.89 | 1.57±0.25 |
| MC | 37.25±2.92 | 0.36±0.06 | 0.16±0.02 | 0.48±0.05 | 4.72±0.75 | 1.56±0.20 |
| PC | 41.88±2.90 | 0.38±0.02 | 0.17±0.03 | 0.47±0.03 | 4.04±0.50 | 1.37±0.19 |
| TBSC | 37.00±2.14 | 0.35±0.04 | 0.11±0.05 | 0.55±0.09 | 4.50±0.69 | 1.60±0.13 |
| TBSCH | 39.13±2.90 | 0.34±0.09 | 0.14±0.06 | 0.60±0.16 | 5.36±0.64 | 1.67±0.32 |

**

**

**Fig. S1 Gel permeation chromatography (GPC) spectra of TBSCH**


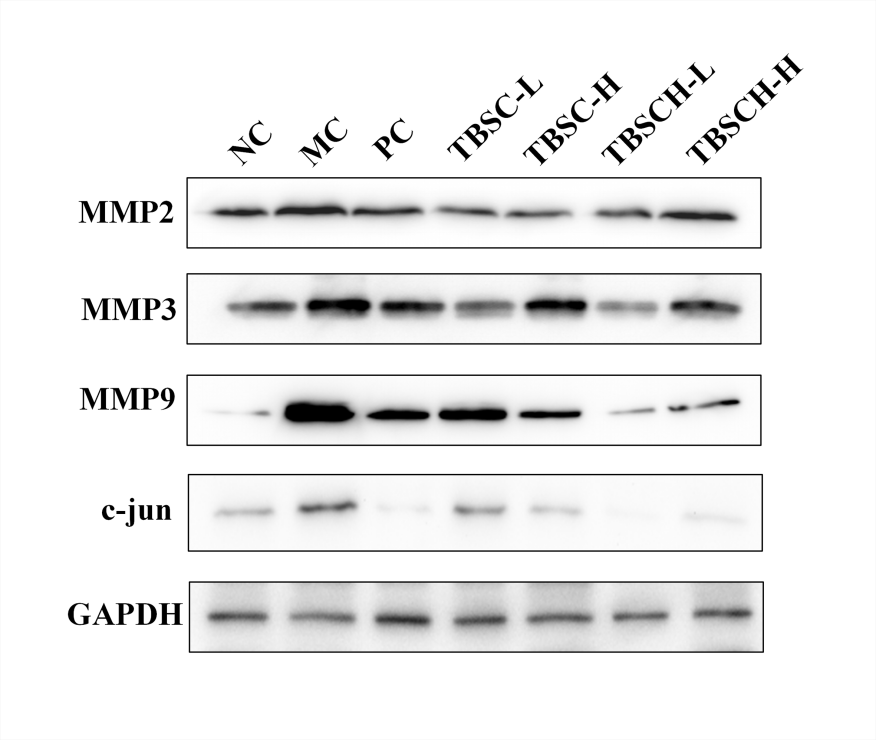


**Fig. S2 Western blotting analysis for mice gavage experiment.** For the mice experiments, we designed seven experimental groups of NC (saline control), MC (UV irradiation alone), PC (ascorbic acid supplementation with UV), TBSC-L (low-dose 50 mg/kg/day TBSC with UV), TBSC-H (high-dose 200 mg/kg/day TBSC with UV), TBSCH-L (low-dose 50 mg/kg/day TBSCH with UV), and TBSCH-H (high-dose 200 mg/kg/day TBSCH with UV).
